# Supplementary figures and images for: Overexpression of GATA5 Inhibits Prostate Cancer Progression by Regulating PLAGL2 via the FAK/PI3K/AKT Pathway
Source: Cancers (Basel). 2022 Apr 21;14(9):2074. doi: 10.3390/cancers14092074 (PMC9099954; doi:10.3390/cancers14092074)

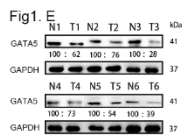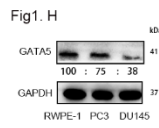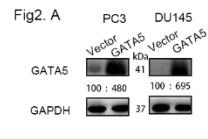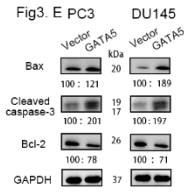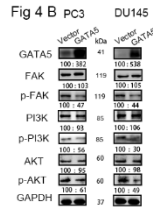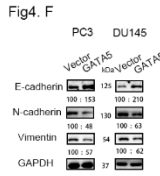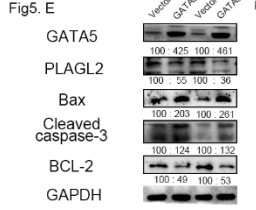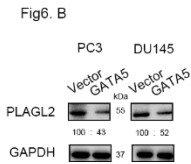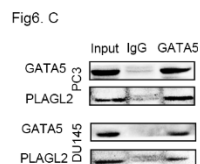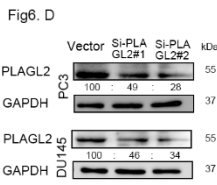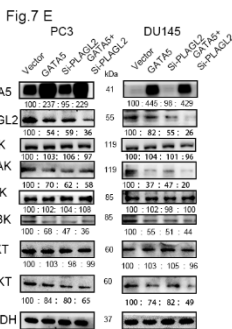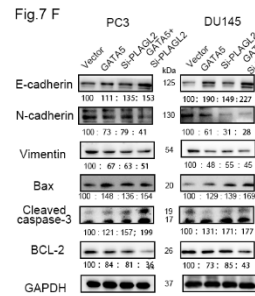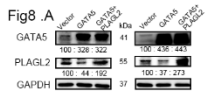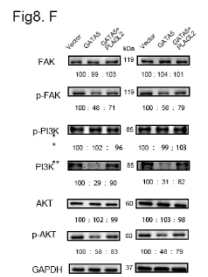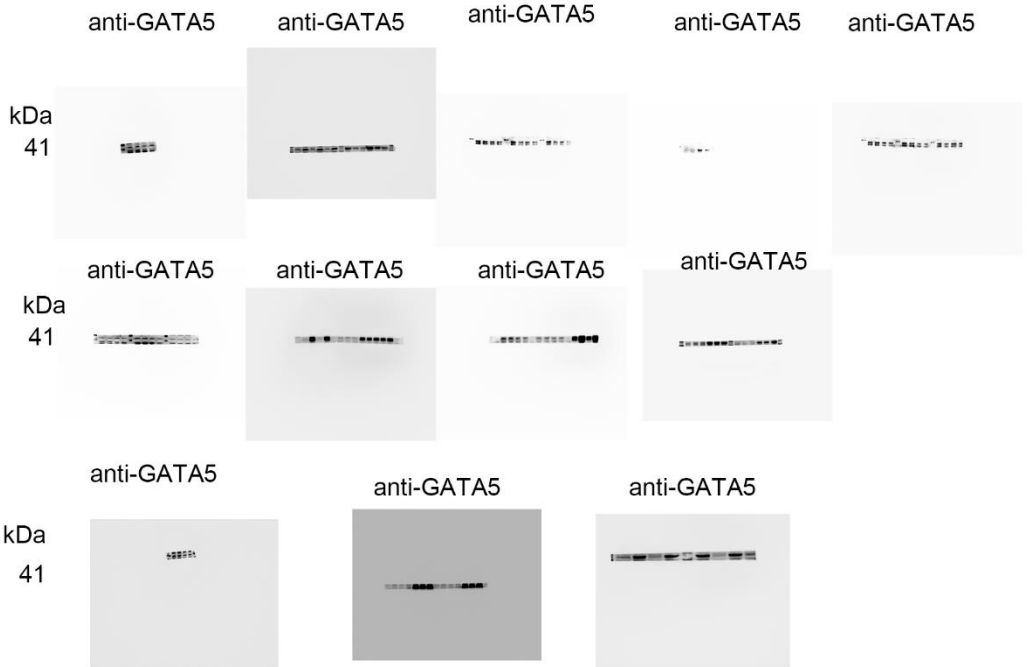

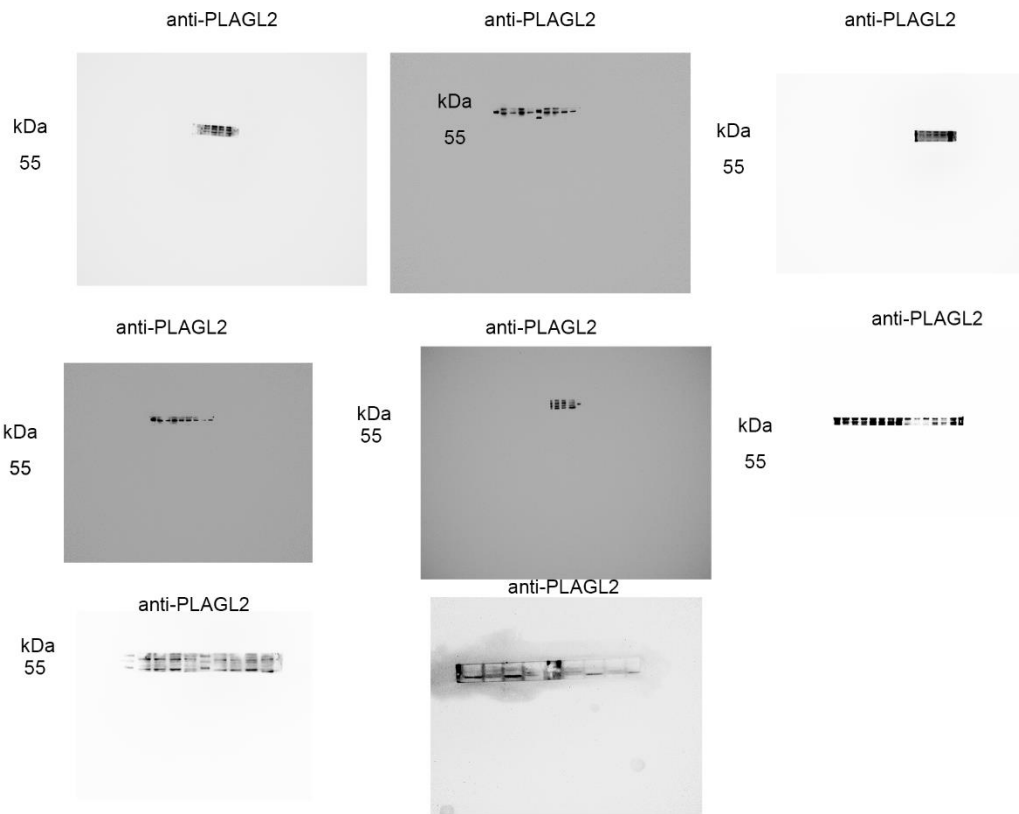

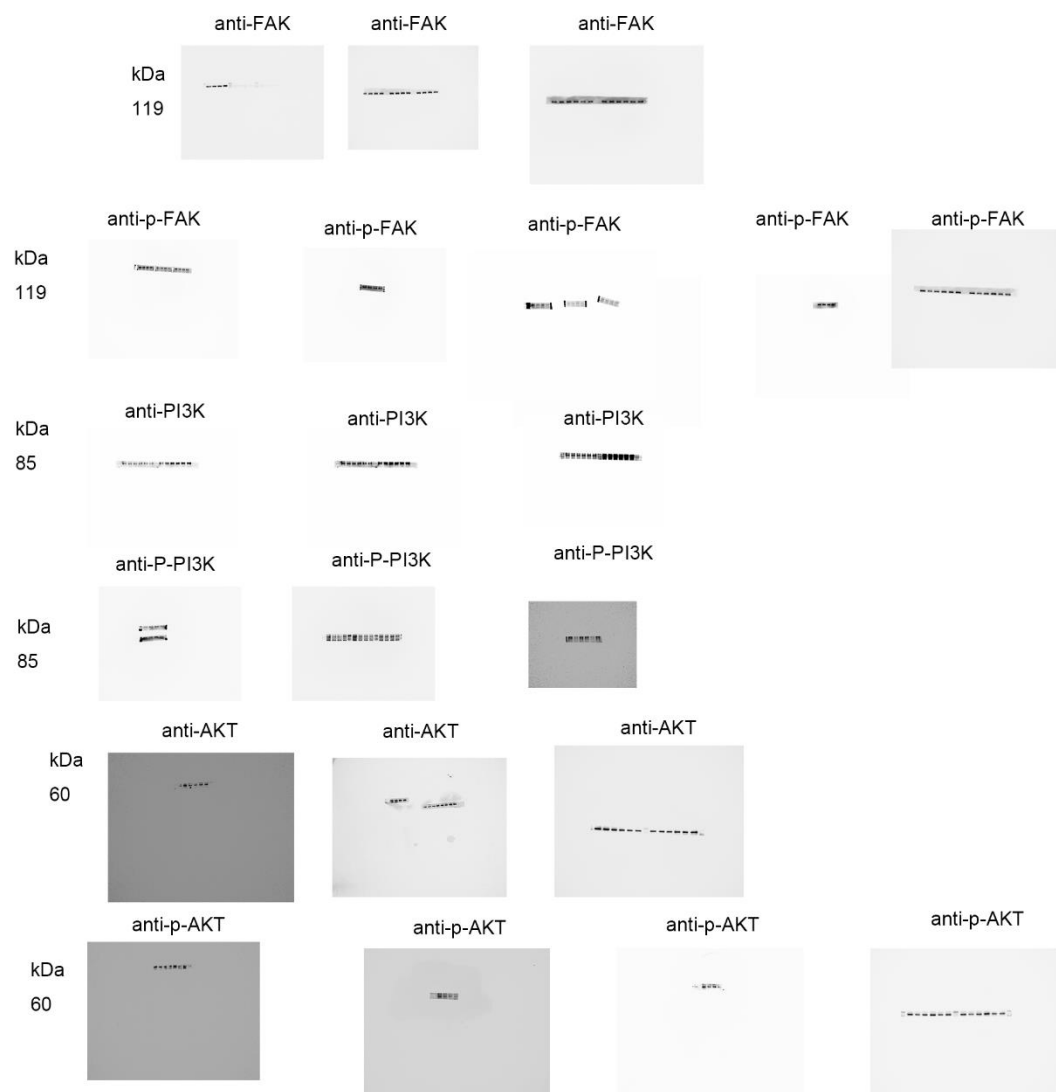

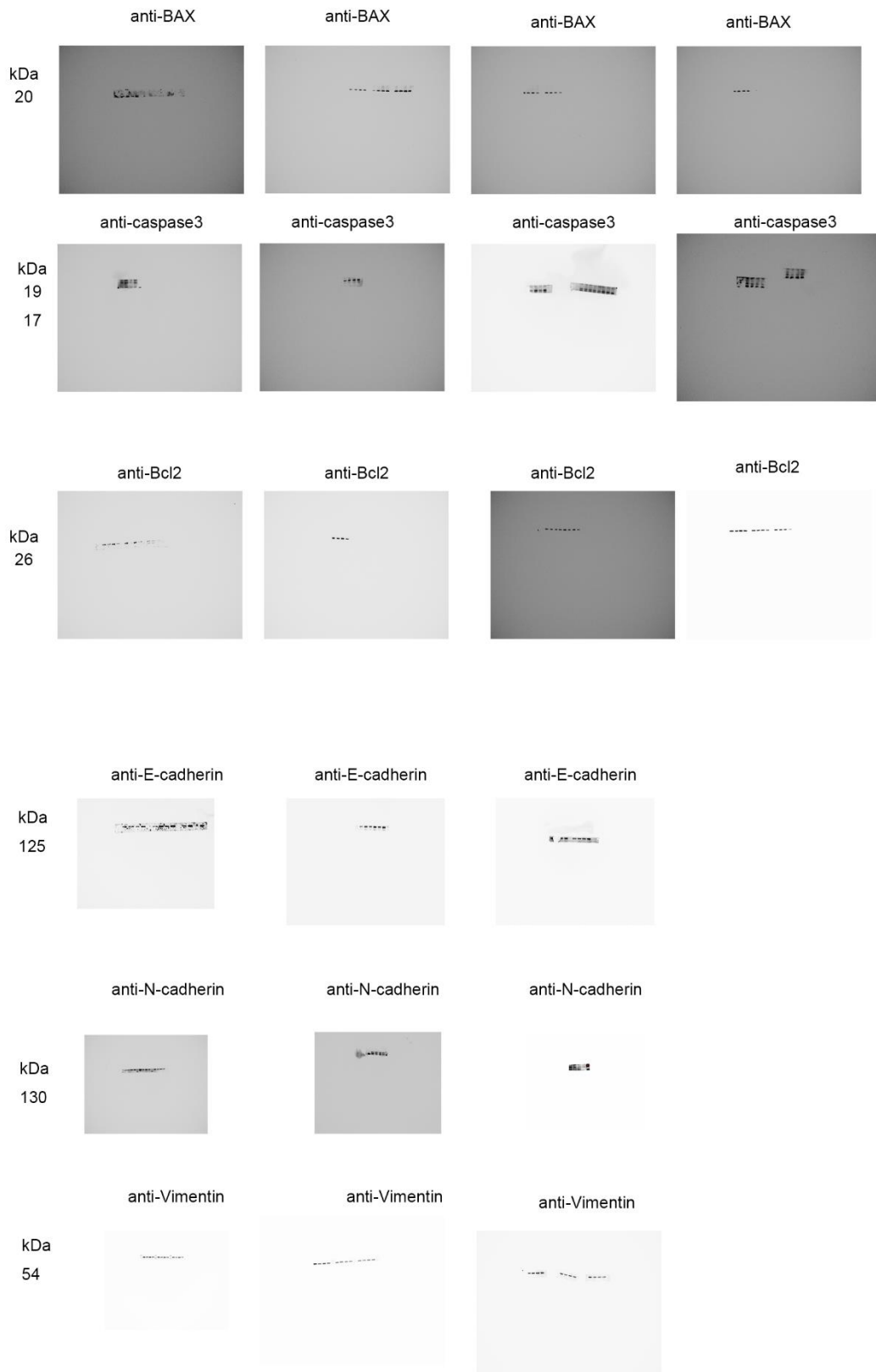

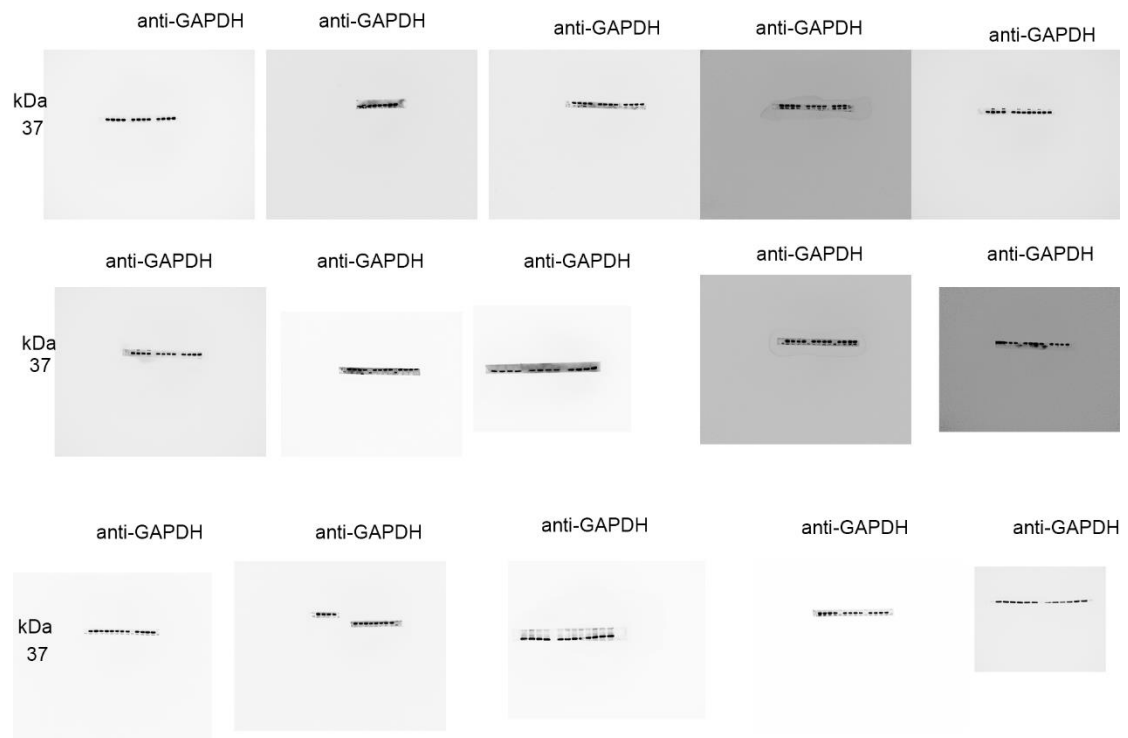

**Figure S1:** Whole western blot in the study.

Supplement: Supplementary file 1 [file cancers-14-02074-s001.zip › Figure S1.pdf]
